# Investigating the impact of preoperative corneal astigmatism orientation on the postoperative spherical equivalent refraction following intraocular lens implantation

**DOI:** 10.1186/s40662-018-0103-4

**Published:** 2018-04-25

**Authors:** Richard N. McNeely, Salissou Moutari, Eric Pazo, Jonathan E. Moore

**Affiliations:** 1 Cathedral Eye Clinic, 89-91 Academy Street, Belfast, County Antrim BT1 2 LS Northern Ireland, UK; 2 Biomedical Sciences Research Institute, University of Ulster, Coleraine, Northern Ireland, UK; 3 School of Mathematics and Physics, Queens University Belfast, Belfast, Northern Ireland, UK

**Keywords:** Biometry accuracy, Prediction error, Corneal astigmatism, With-the-rule astigmatism, Against-the-rule astigmatism, Oblique astigmatism

## Abstract

**Background:**

To investigate the impact of the orientation of preoperative corneal astigmatism on achieving the postoperative target refraction following monofocal intraocular lens (IOL) implantation.

**Methods:**

This study enrolled 339 eyes who had uneventful cataract surgery or refractive lens exchange (RLE) with subsequent monofocal IOL implantation. Eyes were initially categorized dependent upon axial length and then on the orientation of preoperative anterior corneal astigmatism. Group 1 had against-the-rule (ATR) anterior corneal astigmatism, group 2 had with-the-rule (WTR) anterior corneal astigmatism, and group 3 had oblique (OB) anterior corneal astigmatism. The preoperative corneal astigmatism was determined by the IOLMaster (Carl Zeiss Meditec AG). Postoperative refraction was completed for all eyes, and the results were calculated and compared for the separate groups.

**Results:**

In eyes with axial lengths greater than 22.0 mm and less than 25.0 mm there was a significant difference between the magnitude of preoperative corneal astigmatism between groups 2 and 3 with 0.827 ± 0.376 D in group 2, and 0.677 ± 0.387 D in group 3. The mean postoperative spherical equivalent (SE) prediction error was − 0.132 ± 0.475 D in group 1, 0.026 ± 0.497 D in group 2, and − 0.130 ± 0.477 D in group 3. There was a significant difference between groups 1 and 2. There was no significant difference in the magnitude of preoperative corneal astigmatism and postoperative SE prediction error between the anterior corneal astigmatism orientation groups in eyes with axial lengths of less than or equal to 22.0 mm and greater than or equal to 25.0 mm.

**Conclusions:**

The orientation of preoperative anterior corneal astigmatism significantly affected the postoperative biometry prediction error in eyes with astigmatism of 1.75 D or less in eyes with the axial length between 22.0 mm and 25.0 mm. However, the results were not clinically significant.

## Background

Intraocular lens implantation (IOL) following either cataract surgery or refractive lens exchange (RLE) is one of the most commonly performed surgical procedures in the United Kingdom [1]. The modern surgical techniques, including biometry, allows the surgeon to precisely target a postoperative refractive error, and aiming to reduce or eliminate postoperative refractive error is now standard practice [2, 3]. The ability to accurately target postoperative refractive outcomes is mostly dependent on selecting the correct lens power calculated through biometry using an appropriate IOL power formula for the eye under examination. It has been reported that postoperative spherical equivalent (SE) refraction is within ±0.50 dioptres (D) of the target refraction in 75% of eyes following routine cataract surgery [4].

Blurred vision following IOL implantation is a common cause of dissatisfaction, and pre-existing corneal astigmatism can limit the outcomes of postoperative visual acuity. It has been reported that one-third of cataract patients have corneal astigmatism greater than 1.00 D [5]. Preoperative corneal astigmatism is an important factor to consider and affects a surgeon’s choice of IOL, incision placement and whether to utilize peripheral corneal relaxing incisions. The ability to accurately target a postoperative refractive error is now standard practice, and it is therefore essential to understand factors that influence postoperative SE and ultimately the prediction error following IOL implantation. Traditional biometry measures the anterior corneal shape and then utilizes a standardized keratometric refractive index of 1.3375 to define the corneal power. Regression formulas simplify the cornea into a thin lens formula. However, it is known that the overall corneal shape affects biometry. It is assumed that there is a fixed relationship between the front and back corneal surfaces, however it has now been recognized that there is not a fixed relationship between the anterior and posterior corneal astigmatism with both the anterior and posterior cornea changing in shape with age [6–8]. It is unknown how this relationship affects biometry outcomes. Therefore, this study sought to investigate the impact of the anterior corneal shape upon the overall net corneal power effect upon the accuracy of achieving a postoperative SE refraction.

## Methods

Patients included in this study underwent uncomplicated phacoemulsification with IOL implantation. All patients provided informed consent, and all patients gave their informed consent for their anonymised data to be submitted for audit and publication. The Cathedral Eye Clinic Ethics Committee approved this study as an audit study and gave the study the following reference number: CECREC18–02. Preoperatively, the patients were advised of the possible necessity for further corneal laser refractive surgery and the potential risks associated with the operation.

All eyes had 1.75 D or less of preoperative corneal astigmatism, and the eyes were divided, initially, on axial length and then into groups depending upon the orientation of preoperative anterior corneal astigmatism. Eyes with axial lengths greater than 22.0 mm and less than 25.0 mm were classified together. Then, eyes with axial lengths less than or equal to 22.0 mm or greater than or equal to 25.0 mm were considered together. Furthermore, the two separate axial length groups were categorized depending on the orientation of preoperative anterior corneal astigmatism with Group 1 consisting of eyes with against-the-rule (ATR) anterior corneal astigmatism, Group 2 with-the-rule (WTR) anterior corneal astigmatism, and Group 3 oblique (OB) anterior corneal astigmatism.

### Patient assessment

All patients received a full ophthalmologic examination. Biometry was performed using the IOLMaster (Carl Zeiss Meditec AG) and preoperative keratometry results were assessed with the automated keratometer within the the IOLMaster. Autorefraction (OPD-Scan II ARK-10000, Nidek Co., Ltd), subjective refraction (RT-5100 Auto Phoropter Head, Nidek Co., Ltd), uncorrected (UDVA) and corrected (CDVA) distance visual acuities, uncorrected near (UNVA) and intermediate (UIVA) visual acuities, distance-corrected near and distance-corrected intermediate visual acuities, Goldmann tonometry, slitlamp examination, dilated fundoscopy, and retinal optical coherence tomography were completed.

This study included an aspheric monofocal IOL by Rayner Intraocular Lenses Ltd. (C-Flex 970 C). The manufacturer’s A constant is 118.6. The K values, axial length, and IOL power and model were gathered from the IOL Master, and utilizing the optimized lens constants and the SRK/T formula [9] the appropriate target refractive error was chosen. SE refraction in dioptres was calculated postoperatively from the subjective manifest refraction. The deviation of the intended refraction, known as the biometry prediction error, was calculated. Biometry prediction error is defined as the difference between the SE of the postoperative subjective refraction and the target refraction calculated from the preoperative biometry. The prediction error was then compared between the three predefined orientation groups.

### Surgical technique

Surgeries were performed with standard on-axis clear corneal phacoemulsification surgery by the same experienced surgeon (J.E.M). In all cases, the surgery was performed using sub Tenon or topical anesthesia. A 2.75 mm incision was placed on the steepest meridian to prevent the introduction of oblique astigmatism. A 5.00 mm capsulorhexis and implantation of the IOL in the capsular bag was completed in each case.

### Statistical analysis

Statistical analysis was performed using SPSS for Windows software (version 22, SPSS, Inc.) and Excel software (Microsoft Corp.). The Kolmogorov-Smirnov test was utilized to assess normality. The one-way analysis of variance (ANOVA) was applied to compare the outcomes between the different groups in this study. A *P* value of less than 0.05 was considered significant.

## Results

This study included 339 eyes (63.5% female and 36.5% male), with a mean age of 79.5 ± 8.2 years (range 46–95 years).

One-way analysis of variance (ANOVA) was conducted to compare the mean preoperative corneal astigmatism between the groups. Table 1 outlines the comparison of the three orientation groups in eyes with axial lengths between 22.0 mm and 25.0 mm, and Table 2 displays the magnitude of preoperative corneal astigmatism between groups in eyes with an axial length less than or equal to 22.0 mm or greater than or equal to 25.0 mm. There was a statistically significant difference between group 2 and group 3 in preoperative corneal astigmatism orientation with axial lengths between 22.0 mm and 25.0 mm (Tables 3 and 4). Table 5 outlines that there was no significant difference between the magnitude of preoperative anterior corneal astigmatism in eyes with an axial length less than or equal to 22.0 mm or greater than or equal to 25.0 mm.

**Table 1:** Preliminary analysis of the preoperative corneal astigmatism orientation groups. (average axial length eyes, i.e., eyes with axial lengths greater than 22.0 mm and less than 25.0 mm)

Descriptive statistics				
	Mean (M)	Sample size (*N*)	Std. deviation (SD)	Std. error mean (SEM)
Group 1 (ATR)	0.819	88	0.420	0.045
Group 2 (WTR)	0.827	138	0.376	0.032
Group 3 (OB)	0.677	62	0.387	0.049

**Table 2:** Preliminary analysis of the preoperative corneal astigmatism orientation groups. (non-average axial length eyes, i.e., eyes with an axial length less than or equal to 22.0 mm or greater than or equal to 25.0 mm)

Descriptive statistics				
	Mean (M)	Sample size (*N*)	Std. deviation (SD)	Std. error mean (SEM)
Group 1 (ATR)	0.836	20	0.539	0.121
Group 2 (WTR)	0.839	21	0.452	0.098
Group 3 (OB)	0.768	10	0.483	0.153

**Table 3:** ANOVA Table of preoperative corneal astigmatism orientation groups. (average axial length eyes, i.e., eyes with axial lengths greater than 22.0 mm and less than 25.0 mm)

ANOVA Table					
Source	SS	df	MS	F	Prob>F
Groups (Between)	1.052	2	0.526	3.420	0.0340
Error (Within)	43.818	285	0.154		
Total	44.870	287			

*SS*=Sum of Squares; *df* = degree of freedom; *MS* = Mean Square; *F*=F-Statistic

**Table 4:** Pairwise comparisons of preoperative corneal astigmatism orientation groups. (average axial length eyes, i.e., eyes with axial lengths greater than 22.0 mm and less than 25.0 mm)

Pairwise comparison table				
		Mean difference	95% confidence interval for the mean difference	p-value
Pair 1	Group 1	−0.008	[− 0.133; 0.117]	0.987
	Group 2			
Pair 2	Group 1	0.142	[− 0.010; 0.294]	0.074
	Group 3			
Pair 3	Group 2	0.145	[0.009; 0.291]	0.033
	Group 3

**Table 5:** ANOVA Table of preoperative corneal astigmatism orientation groups. (non-average axial length eyes, i.e., eyes with an axial length less than or equal to 22.0 mm or greater than or equal to 25.0 mm)

ANOVA Table					
Source	SS	df	MS	F	Prob>F
Groups (Between)	0.039	2	0.019	0.081	0.923
Error (Within)	11.715	48	0.244		
Total	11.754	50			

*SS*=Sum of Squares; *df* = degree of freedom; *MS* = Mean Square; *F*=F-Statistic

In eyes with axial lengths between 22.0 mm and 25.0 mm the attempted postoperative SE was − 0.29 ± 0.32 D (range 0.35D, − 1.50D) and the achieved SE was − 0.35 ± 0.52 D (range 1.25D, − 1.75D). The mean SE prediction error for the different groups is shown in Tables 6 and 7. There was a significant difference in postoperative SE prediction error between groups 1 and 2 in eyes with axial lengths between 22.0 and 25.0 mm (Tables 8 and 9). There was no statistically significant difference in postoperative SE prediction error between groups in eyes with an axial length less than or equal to 22.0 mm or greater than or equal to 25.0 mm (Table 10).

**Table 6:** Analysis of the postoperative SE prediction error according to the preoperative astigmatism orientation groups. (average axial length eyes, i.e., eyes with axial lengths greater than 22.0 mm and less than 25.0 mm)

Descriptive statistics				
	Mean (M)	Sample size (*N*)	Std. deviation (SD)	Std. error mean (SEM)
Group 1 (ATR)	−0.132	88	0.475	0.051
Group 2 (WTR)	0.026	138	0.497	0.042
Group 3 (OB)	−0.130	62	0.479	0.0609

**Table 7:** Analysis of the postoperative SE prediction error according to the preoperative astigmatism orientation groups. (non-average axial length eyes, i.e., eyes with axial lengths less than or equal to 22.0 mm or greater than or equal to 25.0 mm)

Descriptive statistics				
	Mean (M)	Sample size (*N*)	Std. deviation (SD)	Std. error mean (SEM)
Group 1 (ATR)	−0.056	20	0.650	0.145
Group 2 (WTR)	0.093	21	0.499	0.110
Group 3 (OB)	0.099	10	0.551	0.174

**Table 8:** ANOVA Table of the postoperative SE prediction error of preoperative corneal astigmatism orientation groups. (average axial length eyes, i.e., eyes with an axial length greater than 22.0 mm and less than 25.0 mm)

ANOVA Table					
Source	SS	df	MS	F	Prob>F
Groups (Between)	1.775	2	0.8877	3.749	0.025
Error (Within)	67.479	285	0.2368		
Total	69.255	287			

*SS*=Sum of Squares; *df* = degree of freedom; *MS* = Mean Square; *F*=F-Statistic

**Table 9:** Pairwise comparisons of the postoperative SE prediction error of preoperative corneal astigmatism orientation groups. (average axial length eyes, i.e., eyes with an axial length greater than 22.0 mm and less than 25.0 mm)

Pairwise comparison table				
		Mean difference	95% confidence interval for the mean difference	*p*-value
Pair 1	Group 1	−0.158	[−0.313; −0.002]	0.046
	Group 2			
Pair 2	Group 1	0.001	[−0.190; 0.188]	0.999
	Group 3			
Pair 3	Group 2	0.156	[−0.018; 0.331]	0.089
	Group 3

**Table 10:** ANOVA Table of the postoperative SE prediction error of preoperative corneal astigmatism orientation groups. (non-average axial length eyes, i.e., eyes with an axial length less than or equal to 22.0 mm or greater than or equal to 25.0 mm)

ANOVA Table					
Source	SS	df	MS	F	Prob>F
Groups (Between)	0.278	2	0.139	0.423	0.657
Error (Within)	15.745	48	0.328		
Total	16.023	50			

*SS*=Sum of Squares; *df* = degree of freedom; *MS* = Mean Square; *F*=F-Statistic

**Fig. 1:**
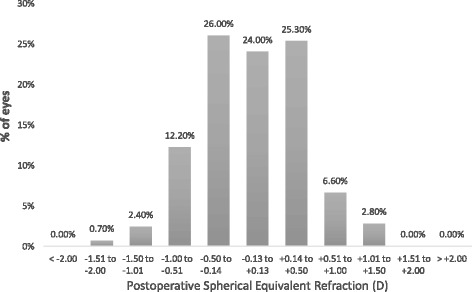
Histogram of postoperative SE refraction relative to the intended target. (average axial length eyes, i.e., eyes with an axial length greater than 22.0 mm and less than 25.0 mm)

**Fig. 2:**
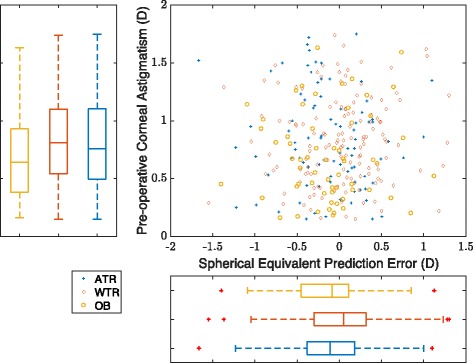
Relationship between preoperative corneal astigmatism and postoperative SE prediction error. Scatter plot representation of the SE prediction error against the value and type of the corresponding preoperative astigmatism (Top right). Boxplot representation of the mean values for each type of the preoperative corneal astigmatism (Top left). Boxplot representation of the mean values of the SE equivalent prediction error for each type of preoperative corneal astigmatism (Bottom right). (average axial length eyes, i.e., eyes with an axial length greater than 22.0 mm and less than 25.0 mm)

**Fig. 3:**
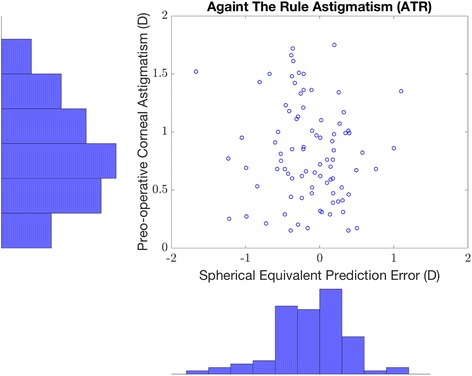
Relationship between the preoperative ATR corneal astigmatism and postoperative SE prediction error. Scatter plot representation of the SE prediction error against the corresponding value of ATR preoperative astigmatism (Top right). Distribution of ATR preoperative corneal astigmatism (Top left). Distribution of the SE prediction error corresponding to ATR preoperative corneal astigmatism (Bottom right). (average axial length eyes, i.e., eyes with an axial length greater than 22.0 mm and less than 25.0 mm)

**Fig. 4:**
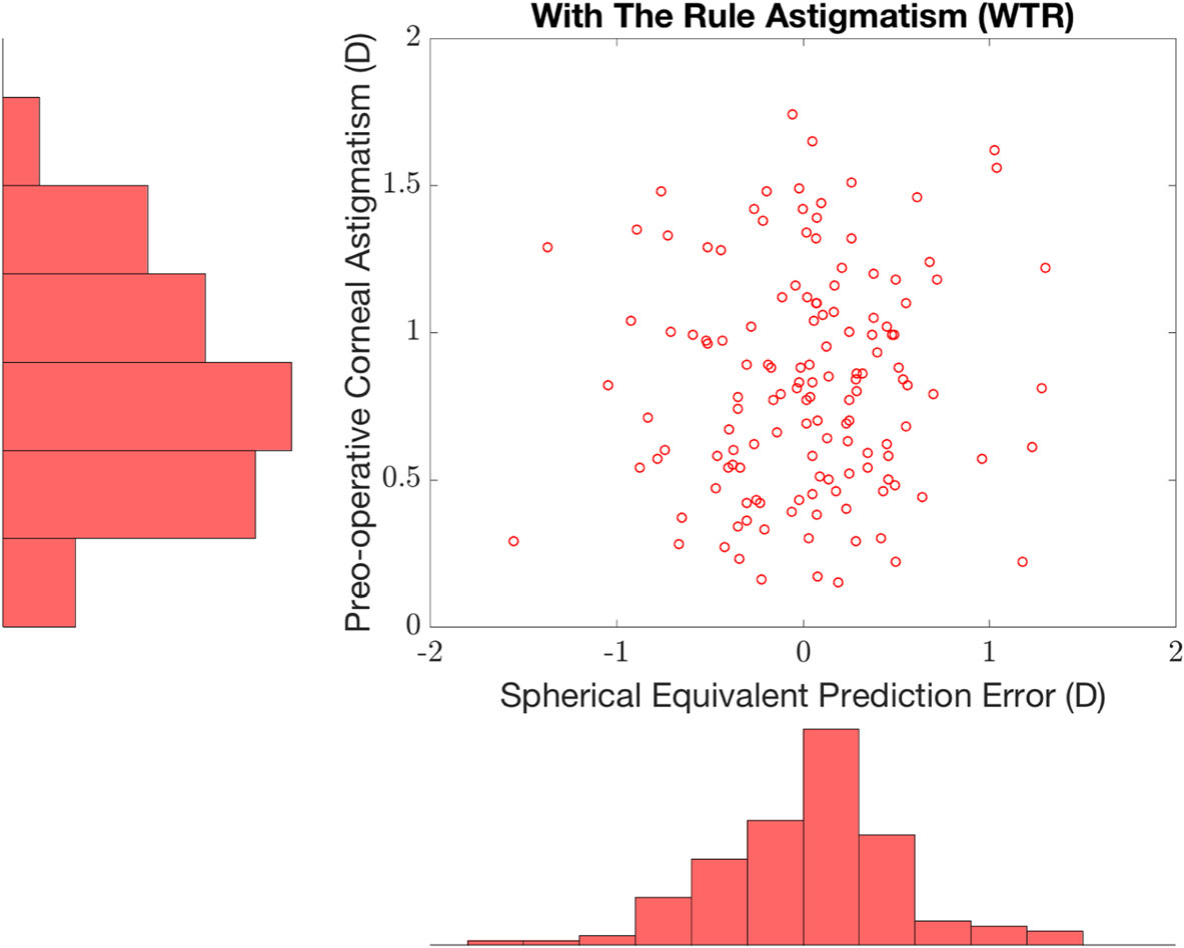
Relationship between the preoperative WTR corneal astigmatism and postoperative SE prediction error. Scatter plot representation of the SE prediction error against the corresponding value of WTR preoperative astigmatism (Top right). Distribution of WTR preoperative corneal astigmatism (Top left). Distribution of the SE prediction error corresponding to WTR preoperative corneal astigmatism (Bottom right). (average axial length eyes, i.e., eyes with axial length greater than 22.0 mm and less than 25.0 mm)

**Fig. 5:**
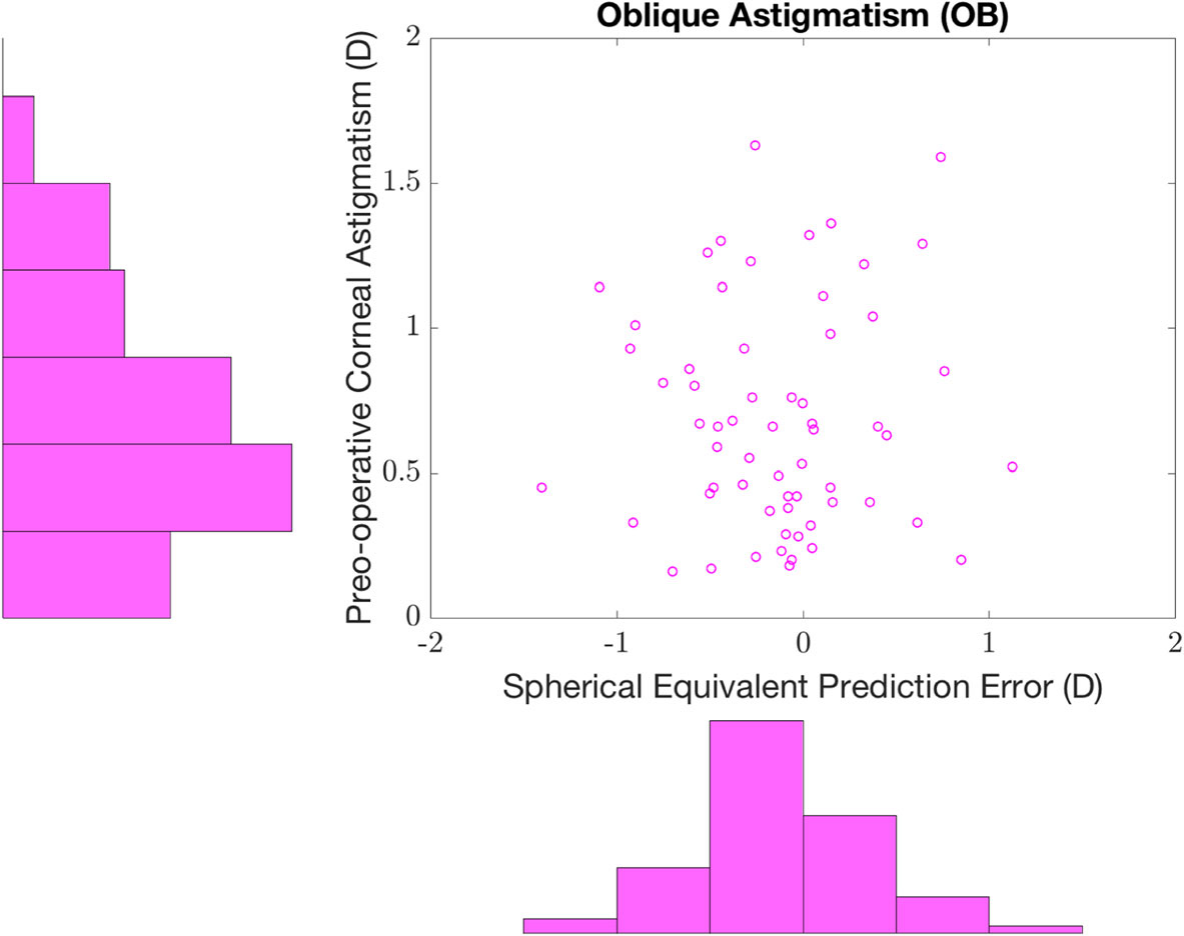
Relationship between the preoperative oblique corneal astigmatism and postoperative SE prediction error. Scatter plot representation of the SE prediction error against the corresponding value of OB preoperative astigmatism (Top right). Distribution of OB preoperative corneal astigmatism (Top left). Distribution of the SE prediction error corresponding to OB preoperative corneal astigmatism (Bottom right). (average axial length eyes, i.e., eyes with an axial length higher than 22.0 mm and less than 25.0 mm)

Figure 1 shows the precision to the intended target refraction where 75.3% were within ±0.50 D and 94.1% within ±1.00 D. Figure 2 displays the postoperative SE prediction error against the magnitude of preoperative corneal astigmatism and Figs. 3, 4 and 5 show the scatter plot representation of the SE prediction error against the corresponding value of the three orientation groups in eyes with axial lengths between 22.0 and 25.0 mm.

## Discussion

The ability to accurately target postoperative refraction is vital in modern cataract surgery and RLE. With advanced surgical techniques, current IOL power calculation formulas and optimized lens constants, a high percentage of patients achieve the target refractive error, with 75% and 95% within ±0.50 D and ± 1.00 D respectively [4]. In this study, we demonstrated that the prediction error was similar to that previously reported with 75.3% within ±0.50 D and 94.1% within ±1.00 D. Studies have been dedicated to investigate the possible factors that may influence achieving the target refractive error, and it has been found that sex, preoperative visual acuity, and glaucoma affected the postoperative prediction error [3]. In an attempt to further understand the factors that may have an impact of the ability to accurately target postoperative SE this study sought to investigate the effects of preoperative anterior corneal astigmatism orientation on the prediction error following IOL implantation. Anterior corneal astigmatism measurements are most commonly obtained with the IOLMaster partial coherence interferometer, which has been found to be highly reliable [10]. The anterior corneal astigmatism measurements are used to calculate the required IOL power and allow a target postoperative refractive error to be determined. This study assessed the orientation of the anterior corneal astigmatism and compared the impact upon postoperative refractive accuracy. In this study, the overall mean anterior astigmatism was 0.79 ± 0.40 D in eyes with axial lengths between 22.0 mm and 25.0 mm, which is similar to that found in a previous study [11]. The mean corneal astigmatism for the three orientation groups is outlined in Table 1, where it was found that the mean preoperative corneal astigmatism was 0.82 ± 0.42 D in group 1, 0.83 ± 0.38 D in Group 2 and 0.68 ± 0.39 D in Group 3 in eyes with axial lengths between 22.0 mm and 25.0 mm. There was a statistically significant difference at level 5% (*p*-value = 0.0340) between the mean preoperative corneal astigmatism for average eyes (i.e., with an axial length greater than 22 mm and less than 25 mm). The pairwise comparison table (Tables 3 and 4) shows that there is a statistically significant difference at level 5% between the mean preoperative corneal astigmatism in Group 2 and Group 3 (*p*-value = 0.033). On the other hand, there was no significant difference between the three orientation groups in eyes with small or more extensive than average axial lengths (Table 5.). Additionally, the mean postoperative SE prediction error was − 0.13 ± 0.48 D in Group 1, 0.03 ± 0.50 D in Group 2 and − 0.13 ± 0.48 D in Group 3 (Tables 6 and 7) in eyes with axial lengths between 22.0 mm and 25.0 mm. The mean prediction error found in another study by Eleftheriadis et al. of 100 eyes was − 0.15 ± 0.38 D [12]. From the results in the ANOVA Table (Tables 8 and 9), there was a statistically significant difference at level 5% (*p*-value = 0.025) between the mean postoperative SE prediction error for the three orientation groups for average eyes (i.e., with an axial length greater than 22 mm and less than 25 mm). The pairwise comparison table (Tables 8 and 9) shows that there is a statistically significant difference at level 5% between the mean postoperative SE prediction error between Group 1 and Group 2 (*p*-value = 0.046). However, there is no statistically significant difference at level 5% between the mean postoperative SE prediction error for Group 1 and Group 3 (*p*-value = 0.999) and Group 2 and Group 3 (*p*-value = 0.089), respectively. Furthermore, the analysis of eyes with axial length less or equal 22 mm or greater or equal 25 mm displays that there is no significant difference in the mean postoperative SE prediction error (Table 10).

To our knowledge, there are no previous studies that explore the impact of the anterior corneal shape on the accuracy of biometry outcomes. It is well known that traditional biometry uses a refractive index of 1.3375 to convert the anterior radius of curvature to a uniform corneal power to overcome the negative effects of the posterior corneal shape. Currently, it is not known if anterior corneal astigmatic changes affect in any way the overall impact upon the ratio of back to front and the net corneal power. This study sought to explore the effect of the anterior corneal shape upon biometry outcomes and therefore the overall net corneal power effect upon biometry outcomes. From this current study, it appears that the anterior corneal shape does not clinically affect the postoperative prediction error.

In our future work, we will further explore the impact of the overall corneal shape on biometry outcomes by utilizing rotating Scheimpflug imaging to directly investigate the effect of the posterior corneal astigmatism on postoperative SE prediction error.

A limitation of this study is the different number of eyes in each of the three anterior corneal astigmatism groups. This was the case because consecutive eyes were recruited to avoid increasing bias by selecting eyes to ensure equal numbers in each of the three groups. Furthermore, as mentioned to be able to make conclusions regarding the overall effect of corneal astigmatism upon postoperative refractive errors analysis of the front and back surface of the cornea is required.

## Conclusions

In conclusion, it emerges that there is a statistically significant difference in the ability to achieve a postoperative refractive outcome in different orientations of anterior corneal astigmatism. However, the overall difference does not appear to be clinically significant.

## Abbreviations

ATR: Against-the-rule
CDVA: Corrected distance visual acuity
D: Dioptres
IOL: Intraocular lens
OB: Oblique
RLE: Refractive lens exchange
SE: Spherical equivalent
UDVA: Uncorrected distance visual acuity
UIVA: Uncorrected intermediate visual acuity
UNVA: Uncorrected near visual acuity
WTR: With-the-rule
